# On Disease Configurations, Black-Grass Blowback, and Probiotic Pest Management

**DOI:** 10.1080/24694452.2023.2289984

**Published:** 2024-02-06

**Authors:** George Cusworth, Jamie Lorimer

**Affiliations:** a Oxford Martin Program on the Future of Food, University of Oxford, UK; b School of Geography and the Environment and Hertford College, University of Oxford, UK

**Keywords:** agriculture, black-grass, disease geographies, integrated pest management, pests, 农业, 黑草, 病害地理, 病虫害综合治理, 病虫害, agricultura, geografías de las enfermedades, manejo integrado de plagas, pasto negro, plagas

## Abstract

This article explores approaches to managing pests that are being developed in response to the faltering effectiveness of antibiotic regimes of chemical control. It focuses on black-grass (*Alopecurus myosuroides*), an endemic plant in European agriculture that has emerged as a serious yield-robber with increasing levels of herbicidal resistance. Following farmers and agronomists who have developed “integrated” approaches to black-grass management, the article identifies approaches to biosecurity that do not target unwanted life so much as they modulate ecological systems in their entirety. Pathogenesis, in this relational understanding, follows not from breaches of dangerous life into healthy space, but from ecological intra-actions that enable the proliferation of some life to compromise the multispecies livability of the body in question. The article contributes to the literature by detailing how this configurational approach works in the world. It traces the polymorphic spatial imaginaries required to map pests well; the process of knowledge intensification needed to reveal which configurations can resist pathogenesis; and the probiotic biopolitical interventions used to safeguard farmland productivity. The article uses black-grass to present a temporal metanarrative of intensive farming causing ecological blowback, leading to the development of approaches to pest management predicated on a pragmatic tolerance toward unwanted life.

## Abstract (zh)

为了应对化学控制中抗生素方法的效力下降, 人们提出了病虫害管理方法。本文对这些方法进行了探讨, 重点是黑草(*Alopecurus myosuroides*)。黑草是欧洲农业的一种特有植物, 由于其抗除草性不断提高, 严重影响了农作物产量。根据农民和农学家开发的“综合”黑草管理方法, 本文提出了生物安全方法——不特别针对不需要的生物, 而是调节整个生态系统。这种关系性理解认为, 病原并非是危险生物闯入健康空间, 而是源于生物扩散危及到多物种生存能力的生态内部活动。本文详细介绍了这种配置性方法。探索了绘制病虫害地图所需的多态空间想象、能够揭示哪些配置可以抵抗病原的知识强化过程、用于保障农田生产力的益生菌生物政治干预措施。本文以黑草为例, 展现了集约化农业造成生态反弹的时间性元叙事, 提出了务实性地容忍有害生物的病虫害管理方法。

## Abstract (es)

Este artículo explora los enfoques en desarrollo para abordar el control de plagas, como alternativa a la poca efectividad que se observa en los regímenes antibióticos de control químico. Se centra el estudio en la hierba o pasto negro, o zacate negro (*Alopecurus myosuroides*), una planta endémica de la agricultura europea que se ha convertido en un serio menoscabo de cosechas, dotado de crecientes niveles de resistencia a los herbicidas. Siguiendo a los granjeros y agrónomos que han desarrollado procedimientos “integrados” para enfrentar la amenaza del pasto negro, el artículo identifica aquellos enfoques de bioseguridad que no se orientan tanto hacia las malezas como a los que modulan en su totalidad los sistemas ecológicos. La patogénesis, de acuerdo con este entendimiento relacional, se enfoca no solo en los brotes de vida peligrosa en el espacio saludable, sino las intraacciones ecológicas que permiten que la proliferación de alguna vida comprometa la viabilidad en multiespecies del cuerpo en cuestión. El artículo contribuye a la literatura al detallar cómo opera en el mundo el enfoque configuracional. Se rastrean los imaginarios espaciales polimorfos que se necesitan para mapear bien las plagas; el proceso de intensificación del conocimiento necesario para revelar cuáles configuraciones puede resistir la patogénesis; y las intervenciones probióticas biopolíticas usadas para salvaguardar la productividad de los campos de cultivo. El artículo usa el pasto negro para presentar una metanarrativa temporal de agricultura intensiva que induce un retroceso ecológico, el cual lleva al desarrollo de enfoques para el control de plagas predicados a partir de una tolerancia pragmática hacia la vida no deseada.

Black-grass (*Alopecurus myosuroides*) is wreaking havoc in the arable areas of England and northwest Europe (Figure 1). It is prolific, highly competitive, and spreads across fields like a black smear. Black-grass produces seed at an astonishingly high rate—as many as 80,000 per square meter—which means that each germination is a hot spot for genetic variation and the emergence of herbicide resistance. Chemical products designed to control black-grass, which themselves take years to develop, become redundant within a few growing seasons. Serious black-grass burdens can reduce yields of staple crops like wheat and barley by as much as 70 percent. One recent calculation suggests that 820,000 tons of wheat are lost in the United Kingdom each year to herbicide-resistant black-grass, costing the country £400 million (Varah et al. 2020). Even for the most hardened farmers, black-grass is referred to in awed, reverential tones. Its name evokes the pestilential, the macabre, and even the satanic.

**Figure 1.**
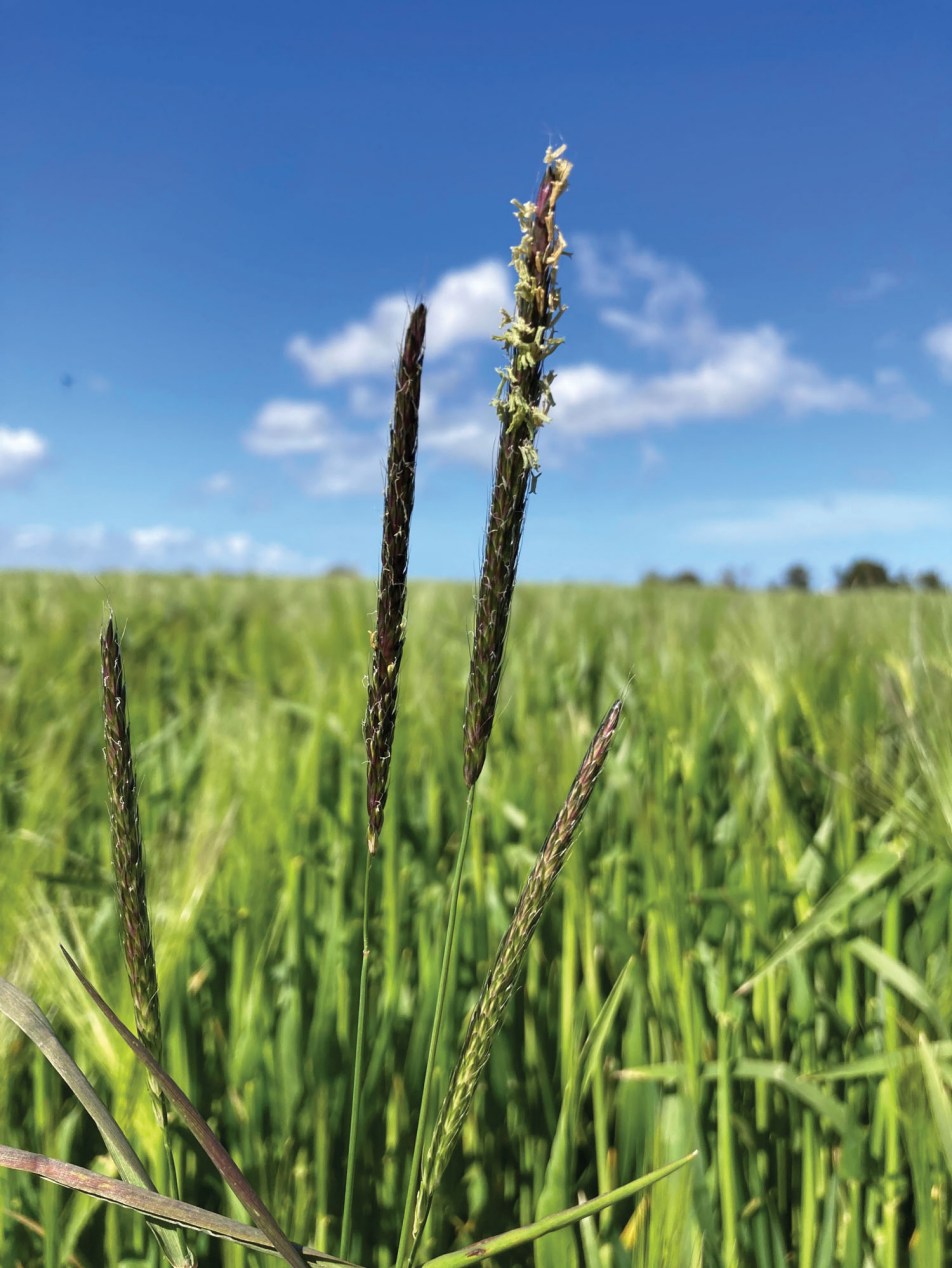
Head of black-grass. *Source:* Image by ETC project at TEAGASC. Reproduced with permission.

The emergence of black-grass as *the* problem weed for certain parts of the farming world illustrates the wider patterns of disease outbreaks originating from—but not necessarily confined to—modern agricultural settings. Geographers and anthropologists have traced how the globalizing monocrop logics that govern intensive food production systems have produced “accelerator landscapes” (Gandy 2022) whose ecological simplification and space–time compression enable the proliferation of viruses, fungi, bacteria, and plant and animal pests (Gandy 2023).

Geographers researching the emergence of such pathogenic life have called for a change in how we map the topologies of plant, animal, and zoonotic disease: away from bounded regions threatened by external contamination, toward the networks of ecological relations that allow pathogenesis to take root (Hinchliffe et al. 2013; Hinchliffe et al. 2016). The emergence of disease, in this latter understanding, is not solely the outcome of a breach of dangerous life into an otherwise healthy space. Instead, it is immanent from the ecological milieu in question (Barker 2015; Lorimer 2017). Complex “intra-actions” (following Barad 2007) between multiple lives folded together permit or frustrate a pathological event. An outbreak happens when a configuration is such that it allows the proliferation of some life to come at the expense of the others it might have otherwise lived alongside. This shift in understanding necessitates a change in the language used to describe health and disease: from breaches to tipping points, from borderlines to borderlands, from external to endogenous, from inherent to relational, and from pathogen-free to immuno-competent. In this article, we refer to these changes as a shift from a *contamination* to a *configuration* model of health, pathogenesis, and disease (after Hinchliffe 2015).

The literature on these topics has largely been written in a diagnostic and critical register. Scholars have shown how the contamination model underpins attempts to keep desirable life (like crops and livestock animals) at hygienic spatial remove from those with pathogenic potential through high-tech infrastructure and biosecurity protocols (Hinchliffe et al. 2016). They show how the success of this approach is thwarted by the way these simplified environments create, rather than displace, the conditions needed for pathogenic proliferation (Tsing 2017; Chao 2022). We add to this literature by detailing the principles of a configurational approach that seeks to manage the pathologies immanent to intensive agriculture.

After we describe the methods used in the production of this article, we review the literature on disease geographies and provide an introduction to the black-grass weed at the heart of the research. We then describe integrated pest management (IPM)—an approach that embodies the configurational mode of pest and disease control—and introduce the practices deployed to mitigate against black-grass. In the main analytic section, we use the example of black-grass to (1) map the polymorphic spatial imaginary needed to understand agricultural pathogenesis as an outcome of agro-ecological relations; (2) document the “intensive,” “holistic” and “contextual” knowledge practices required to know and manage disease as an outcome of an ecological configuration rather than the product of a contamination event; and (3) examine an emerging, probiotic model of “agribiopolitics” (Hetherington 2020) manifest in IPM strategies. These address the problem of black-grass by modulating agro-ecological relations rather than isolating and destroying the unwanted life itself. They are premised on a pragmatic (if reluctant) tolerance toward unwanted life. In conclusion, we propose a metanarrative for agricultural biosecurity and flag its implications for geographical scholarship developing ways of living well with disease, awkward plants, and other pests (Ginn 2014; Giraud 2021; Argüelles and March 2022).

The novelty of the article lies not in identifying the explanatory deficiencies of contamination models of disease. Similar claims have been made in both agricultural (Hinchliffe et al. 2016) and epidemiological contexts (Rosenberg 1989) for some time. Nor is it unique in its attempt to claim that an industry-wide shift is underway (although as chemical controls cease to work, these interventions really are becoming more popular). Instead, the article’s contribution can be found in the way it attends to the details of how a configurational approach to arable pests and disease works out there in the world. The article asks the following questions: How can the relational aspects of a disease or pest outbreak be conceptualized when they are so complex, dynamic, and messy? How can appropriate and actionable knowledge on the situation be produced? What do the resulting strategies end up looking like?

## Methods

Our analysis is grounded in interviews, farm visits, and ethnographic observation of industry events in the arable lowlands of southern and eastern England. We focus first on farmers (thirty-five interviewees) and second on researchers, agronomists, and farm advisory professionals dedicated to pest management and black-grass control (eight interviewees). For both groups, interviews were a mix of telephone and face-to-face conversations.

With regard to the project’s data collection, there was a chronology to how these two groups were consulted. Interviews were initially conducted with the first group: those managing commercial agricultural businesses (selling commodities into large markets rather than direct-to-consumer niches), medium to large in size (∼150–12,000 ha), operating mixed or arable systems, and those experimenting with IPM, regenerative practices, or both. These farmers were contacted through a research project on regenerative agriculture (Cusworth et al. 2022) and crop diversification strategies (Cusworth, Garnett, and Lorimer 2021a, 2021b). The ubiquity of the black-grass problem was such that, among these farming actors, it was a recurring topic of conversation—noteworthy either for the toll it was taking on the farm business, or because of the unlikely successes that were being recorded in its mitigation.

After initial rounds of analysis, and after the black-grass weed emerged as a coherent theme, several repeat interviews from the first group were conducted. Contact was also made with actors in allied professions—those that made up the second group of interviewees. This latter group spoke from positions in agronomy firms, extension services, and agricultural research stations. They added a level of research expertise to the farming interview data. In different ways, this latter group verified, contradicted, and complicated the reports from the agricultural front line. The project thus followed inductive and purposeful methods. An overarching research project (on regenerative agriculture and crop diversification) led to a more specific engagement (black-grass), which, in turn, generated a need to seek out a specific set of research participants.

Our data capture also included textual analysis of pest management research, technical guides written for farmers, and industry communications. This helped confirm that the practices and ideas we encountered during interviews had broader traction in the industry. We refer to these materials throughout the article. Speaking to our agronomy experts late in the data collection process also allowed us to get a sense of how the perspectives of our farming participants compared with the more strategic vistas these experts had on the sector. Once we had finished our article, it was returned to a small selection of our interviewees (six, from both the farming and agronomy cohorts) for corroboration. Although they admitted that our terms and concepts were novel to them, and some suggested minor edits, all agreed with the sentiments and conclusions we reached.

## Mapping the Geographies of Biosecurity

Geographers have demonstrated how conventional biosecurity—“the protection of production systems from the threats caused by pests, pathogens and diseases” (Ilbery 2012, 310)—is predicated on a Pasteurian, germ-theory model (Paxson 2008). This holds that bodies, landscapes, and populations are healthy until their borders are invaded by hazardous life. To guard against deleterious health outcomes, the borders of safe life must be policed to repel whatever breaches might otherwise compromise its good functioning (Hinchliffe et al. 2016). They suggest that this model is founded on a “regional” (Mol and Law 1994) or “topographic” (Hinchliffe et al. 2013) imaginary in which space is divided into discrete and bounded territories.

This conceptualization, which predominates in public, biomedical, and biosecurity thinking, is being complicated by an emerging understanding of bodies as composite amalgams: ecological assemblages whose good health necessitates rather than precludes the presence of other species (Barker 2015; Braverman 2022). New and established research in microbiology, immunology, and disease ecology is refiguring health as a multispecies achievement premised on ecological intradependencies (McFall-Ngai et al. 2013; Faure, Simon, and Heulin 2018). It suggests that plants and animals are holobionts, composed of, and ontologically inalienable from the collegial ecologies of microscopic bacteria, fungi and viruses, and inert matter from which they are composed. Many of these nonhuman lives, whose presence makes life livable, are symbionts. These scientists diagnose widespread “dysbiosis” manifest in the rise of autoimmune, allergic, and inflammatory disease as well as in the proliferation of drug-resistant organisms, caused in part by the absence of the symbionts that once configured holobiont ecologies (Blaser 2014). This postgenomic ecological ontology has become popular in the environmental and medical humanities (Lorimer 2016) as part of a more general decentering of the human in accounts of the distribution of agency, intelligence, creativity, and ethical value in a more-than-human world (Brives, Rest, and Sariola 2021). Here, nature no longer acts as a passive backdrop for the activities of self-contained human bodies. It has its own world-making vitality, and we are entangled within it.

This relational disease ontology helps explain how intensive monocrop systems (Tsing 2017; Morand and Lajaunie 2021) and livestock feedlots (Blanchette 2020) provide breeding grounds for pathogenic proliferation. Holobiont ecosystems, which are deprived of symbionts in the service of expediting the growth of a target crop or animal, are thrown into dysbiosis, leading to the emergence and unruly spread of infections and disease (Perfecto, Jiménez-Soto, and Vandermeer 2019). With the diminishing efficacy of fixes predicated on antibiotic rationalization, the costs involved in maintaining control over volatile monocrop landscapes are beginning to outstrip the profits they generate (Guthman 2019; Giraud 2021).

Responses to these problems range in scale and type, but an important subset share a commitment to fostering health through the presence, not absence, of life. Documented examples of this “probiotic turn” (Lorimer 2020) include consumers concerned with the adverse health impacts of modern pasteurized diets who are seeking out unpasteurized foodstuffs for the bacteria, yeast, and mold they contain (Paxson 2008); contract livestock farmers covering their sterile pigs in muck when they arrive on the farm to acclimatize the animals’ immune systems to the messy and complex multispecies environments they will have to inhabit (Hinchliffe and Ward 2014); and farmers in Indian Natural Farming systems fermenting the manure of their livestock animals before applying the preparations back onto the land to improve soil health (Münster 2021).

These probiotic strategies are founded on a radical reconceptualization of the geographies of health and disease (Lorimer 2017). Moving away from the contamination model, these actors grapple with how the configuration of ecological relations structures the dynamics between interwoven and folded multispecies life. Salutary and violent outcomes are, in this configurational mode, the product of how relations unfold in space and over time, how many different things there are, how many of each thing there is, how they move, how they interact, and so on. Health and disease become relative, not absolute, terms (Hinchliffe 2022a), applicable to ecosystems, landscapes, and other multispecies bodies rather than the discrete lifeforms within them.

Far from the grand narratives of dangerous life besieging healthy space, here health and disease emerge from mundane encounters between an array of more-than-human, ecological, political, economic, architectural, and social factors (Brown and Kelly 2014). Building on this work, this article aims to codify the key components of a post-Pasteurian, postcontamination, proconfiguration model of control. It explores how different and potentially antagonistic lives are made to live alongside one another, and it describes how such strategies work in the messy realities of agricultural management.

We see a clear and varied demand for this scholarship. Ginn and colleagues have noted the way scholars have averted their gaze from multispecies entanglements “when togetherness is difficult, when vulnerability is in the making, and death is at hand” (Ginn, Beisel, and Barua 2014, 114). Giraud et al. (2019) called for attention to be directed toward “ways of ‘being alongside’ life that is difficult to live with” (357) in a manner that learns from a history of failed technofixes. In developing their analysis of the patchy Anthropocene, Tsing, Mathews, and Bubandt (2019) called for research attuned to the configurations that might enable multispecies resurgence. We offer this article in response to these calls.

## The Emergence of Black-Grass as a Pest

Black-grass is a plant endemic to Britain, France, Italy, Germany, Denmark, and Holland. It was noted as a weed with potentially troublesome impacts for English agriculture nearly 200 years ago (Sinclair 1838), but it is only in the last few decades that it has become one of, if not *the,* worst problem weeds for arable farming in northwest Europe (Riches 2022). Its frontiers are expanding, too, with pernicious black-grass problems emerging in China, the United States, and Russia, and herbicide-resistant populations documented in Czechia, Poland, and Sweden (Moss 2017).

As with other weeds (Barua 2023), the contemporary abundance of black-grass results from a broader history of land-use change. Three interlinking causal factors stand out: (1) the intensification of agricultural management; (2) the deterioration of the ecological conditions of the land, especially soil health; and (3) the difficulties of managing black-grass with chemical controls, particularly due to the emergence of herbicidal resistance.

In relation to the first driver, agricultural management in the United Kingdom, Europe, and beyond, underwent dramatic shifts in the postwar period. With the emergence and increasing affordability of synthetic pesticides and fertilizers, the crop rotations and mixed arable-livestock land uses that were historically used to improve fertility and combat pests became of limited agricultural value (Cusworth, Garnett, and Lorimer 2021a). This had several important consequences for black-grass. Paired with developments in frost-resistant seed cultivars, mainstream farming witnessed a rise in winter cropping, the loss of spring cropping, and the specialization of farms into either arable or pasture systems. Where farmers once left their fields fallow after a late-summer harvest, or where they planted grass mixes or left crop stubble to be grazed by ruminant animals like sheep and cattle before sowing the following year (spring cropping), farmers now typically get successive cash crops in the ground immediately after harvest (winter cropping). This continuous cycle requires additional tillage in the September and October period, which is when black-grass seed germinates. This means that if there is seedbank in the soil, it will germinate and, in turn, compete with the crop for light, nutrients, and water. These changes are part of the “intensification” of agricultural management.

The second factor is soil degradation. The simplification of crop rotations, the loss of fallowing and grass-arable cycles, and the increasing intensity and depth of tillage programs have degraded soil ecology and increased topsoil compaction (Arriaga, Guzman, and Lowery 2017). These processes affect soil drainage: There are fewer worms and other invertebrates to aerate the soil, simpler root systems to break it up, more tillage disrupting the establishment of soil ecology, and more passes of heavy machinery compacting it. Black-grass thrives in moist conditions and is very competitive in the poorly drained soils being produced by intensive farm management (BASF 2018).

Finally, herbicide efficacy and resistance play a role. Black-grass is a grassweed, not a broadleaf weed, and is taxonomically very close to many of the staple plants (like wheat and barley) of contemporary arable production in the United Kingdom and Europe. This genetic overlap has helped make it difficult to manage. As the growth mechanisms and genetic makeup of black-grass are similar to the crops being grown, it is hard to develop selective herbicides that can be applied to the field after the weed has emerged in the crop. Furthermore, black-grass’s genetic development pathways and its high seeding rate make it particularly quick to develop resistance to selective herbicides (Cai et al. 2021). As excess chemical applications select for more resistant weeds, the emergence of herbicide resistance is correlated with the intensity of historical chemical control programs (Hicks et al. 2018). With companies increasingly reluctant to pour significant research and development budgets into such testy conditions, the products for black-grass now entering the market are typically based on compounds that were shelved in earlier rounds of product testing due to their limited efficacy.

## Integrated Pest Management

To counter troublesome pests like black-grass, our interviewees are experimenting with a range of practices that can be grouped under the rubric of IPM. We use this term as our interviewees were happy to apply it to their practice, albeit often in conjunction with other terms like organic, regenerative, and agroecological. Some observed strict IPM practices, and others used its principles and research as a more general set of management heuristics.

IPM aims to integrate the goals of pest management into practices of food production and landscape management so that pest suppression becomes an emergent feature of the farm’s agro-ecosystem rather than something applied to it in chemical form. IPM seeks to reduce, as far as is practicable, the use of herbicidal, insecticidal, and fungicidal products (Naranjo 2001). This need not entail their total exclusion as it would in an organic system, but “chemical controls” are the final level in the IPM flow of prioritization—the measure of last resort.

IPM stresses the importance of “cultural” controls. These refer to the agricultural practices that configure the material conditions of the farm, including the ecologies of insects, animals, and bacteria, as well as biophysical factors like moisture content, soil structure, and drainage. It aims to prevent or combat disease events and pest outbreaks by creating a set of agro-ecological relations that are hostile to pathological proliferation. It does this via intervention in the agricultural activities that configure those relations: crop rotations, the presence or absence of livestock animals, fertilizer application programs, tillage regimes, biodiversity areas, water quality, hedgerows, buffer strips, and so on. IPM takes as its unit of intervention the entire agro-ecosystem of a farm, as opposed to the pest itself (Deguine et al. 2021).

IPM is based on the calculation of *thresholds*. Under this rubric, it is only when some pest or disease burden becomes sufficiently problematic that chemical applications are permitted. Before these thresholds are met, managers must prioritize cultural methods of control and, more generally, learn to live with the pests that could potentially cause them and their businesses harm. We discuss the biopolitical implications of this tolerance later.

**Figure 2.**
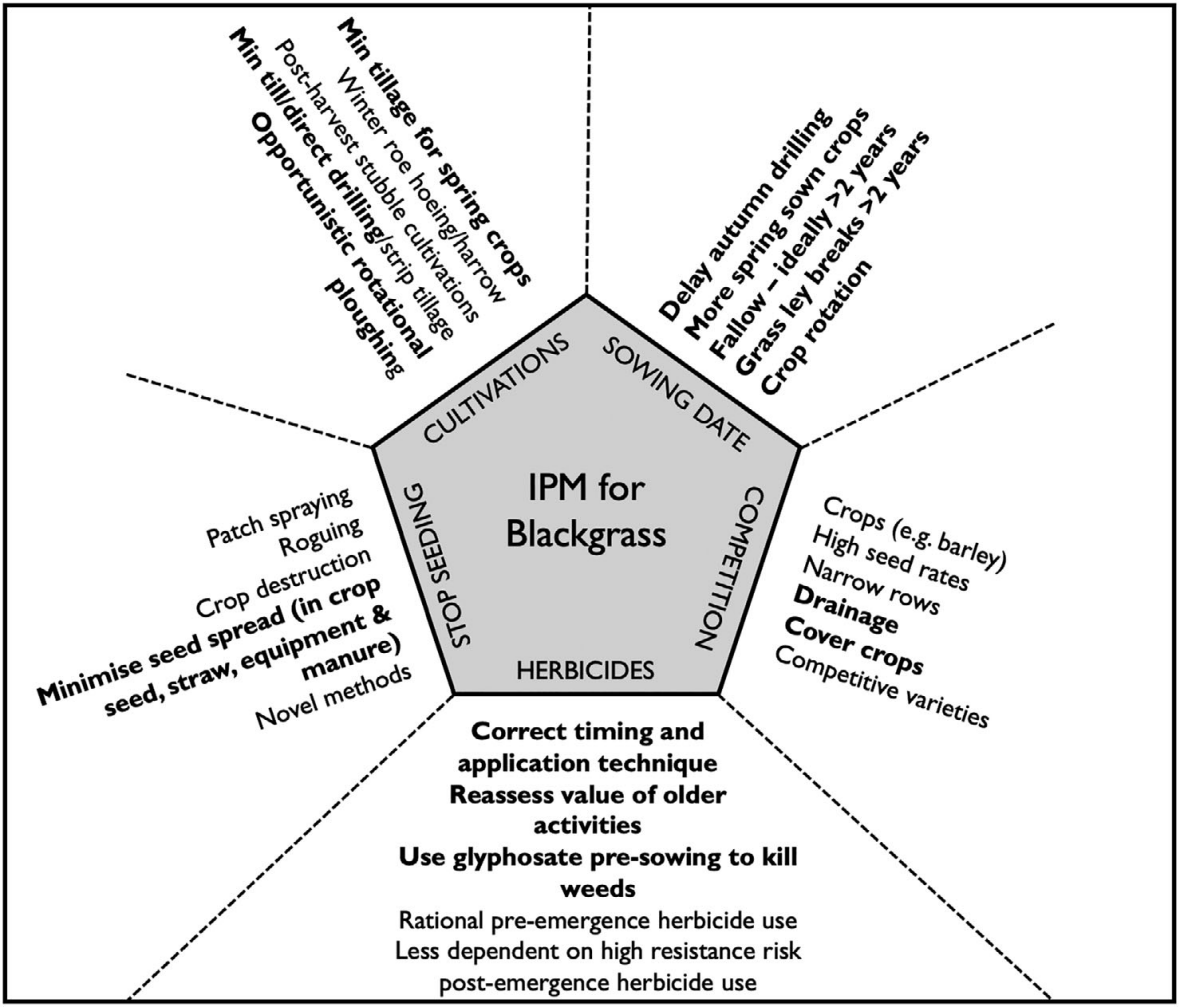
Integrated pest management (IPM) strategies for black-grass management. The practices in bold are those we discuss in the analysis. *Source:* Adapted from Moss (2017).

Figure 2 provides an overview of the IPM strategies used for controlling black-grass, which we discuss in more detail in the analysis that follows. The most important factor in IPM approaches to black-grass management is the reduction of soil disturbance in September and October to prevent any latent seedbank in the soil from having the opportunity to germinate. This typically entails a shift from winter to spring cropping: sowing arable cash crops in February, March, or April (or later, depending on the crop) rather than drilling the seeds directly after harvest in August, September, or October. There are several land management practices that enable this transition. The establishment of cover crops—like vetch, mustard, and clover that are grown to keep the soil covered, and to improve its nutrient content and structure—provide an alternative agronomic benefit to make up for the lower yields of spring-sown crops (which enjoy a shorter growing period than those sown in winter).

Livestock-arable integration is also seen as a key tool for black-grass control (Hill 2015). Grazing animals, particularly cows and sheep, allow farmers to introduce multiyear grass breaks from the cropping cycle. This helps build soil structure and improve drainage. These grass “leys” can even keep the black-grass seed buried for long enough that it becomes inactive. Livestock animals can also be fed on the cover crops grown over the winter, which provides an additional financial return from a move to spring cropping.

## Analysis

In the following sections, we examine three central features of the configurational model of pest management that are illustrated by our UK black-grass example: its spatiotemporal imaginary, its process of “knowledge intensification,” and its probiotic mode of agribiopolitics. These three reflect the *ontological, epistemological,* and *ethico-political* consequences of a configurational approach to weed control.

### A Polymorphic Spatiotemporal Imaginary

In this section, we tease apart the different elements of the “polymorphic” (after Jessop, Brenner, and Jones 2008) spatiotemporal imaginary that is helping our research informants conceptualize pathogenesis as immanent from the configuration of agro-ecological relations. Over the course of our interviews, we found that our participants reconciled and overlayed three different topologies onto the bounded regional map of disease we introduced earlier. A black-grass outbreak certainly has a bounded territorial aspect: An outbreak on the farm could be drawn on a two-dimensional cartographic map, and efforts must be made to stop the weed from moving out. What is missing, though? What ontological additions are being made by those following IPM principles to better understand how, why, where, and when it spreads?

To answer these questions, we must start with the common outbreak narrative our farmers offered us when discussing black-grass (Wald 2008). Tom Sewell, an arable farmer who had completed a Nuffield Scholarship on no-tillage and regenerative practices, explained:

Machinery, insects, birds, the wind … there’s a hundred and one different ways it moves about.

These same ideas can be seen in the left-most portion (the text that reads “minimize seed spread in crop seed, straw, equipment, and manure”) of Figure 2—our simplified version of an image that circulates on Web sites and forums dedicated to black-grass management (e.g., Agricology 2020). This is the common starting point for black-grass management: To mitigate against black-grass you need to know how it moves, and to understand how it moves you need to think in terms of vectors of networked connection. Like the webs of global trade, these material linkages are disjointed, rhizomatic, and far-reaching. Many can be accounted for and acted on. For example, to stop black-grass from moving between fields and farms, industry press articles encourage readers to clean machinery after it is used in a field with high levels of the weed and to vet their contractors to ensure they do the same (Crop Production Magazine 2017). They also encourage farmers to be vigilant about where they buy their manure to ensure they are not importing black-grass seed from heavily burdened farms (Irish Farmers’ Journal 2022). To mobilize a configuration-based approach to pathogenesis, our actors first overlay a model of networked connection onto the “bounded space” understanding of health and disease.

Not all of vectors of black-grass movement can be mapped in this same strategic way, though. The distribution of black-grass seed by the wind and birds amounts to a more random and potentially totalizing mobility. Clearly, though, black-grass burdens are not evenly spread. Some landscapes, farms, and fields have proven themselves more vulnerable to outbreak than others. Given the constant circulation of potentially dangerous pests and diseases, good health is not so much about presence or absence but rather an immunocompetent ability to live with other life (Hinchliffe et al. 2013). To better account for and act on these dynamics, our farmers call on what we term a *hot spot* imaginary.

Consider the following excerpts. When asked about the conditions that are likely to lead to a black-grass outbreak, Stephen Briggs, a researcher and advisor at Innovation for Agriculture*—*a research and agronomy firm with a focus on sustainable farming solutions—explained:

Black-grass likes and thrives in conditions of poorly structured, anaerobic soils, with low levels of organic matter, low aeration, consolidation, compaction. … No farmer has a black-grass problem. Farmers have a soil problem.

The BASF handbook on black-grass management also stresses the correlation between soil quality and black-grass risk. It recommends spatially targeted applications of soil amendments to biophysically reshape the configurations of at-risk areas:

Soils with a high clay content and poor structure, and soils that maintain high water content, will favour black-grass. To assist cultivation practices designed to improve black-grass control, soil physical conditions can be improved by applications of organic matter and Gypsum. (BASF 2018, 1)

Here, the pathogenicity of black-grass is a function of the ecological configurations (mostly, but not exclusively, those of the soil) in which it exists: what things there are in a farm’s agro-ecosystem, how many of those things there are, and how they relate to one another over time. The hot spot imaginary helps farmers conceptualize how ecological dynamics determine why some fields, farms, and landscapes are particularly prone to an outbreak, and it allows them to make spatially tailored black-grass management decisions.

From this vantage point, a pest outbreak is not extrinsic to the farm’s agricultural milieu, but “are part and parcel of [the] play of forces” (Hinchliffe 2022b, 147) of which it is constituted. Although terms like *invasion* and *contamination* are meaningful in IPM, notions of health and disease are more often used to describe the agro-ecological conditions of a given space. Black-grass becomes a sentinel, symptomatic of ecosystem dysbiosis (e.g., poor soil condition) rather than the manifestation of a discrete pest. Mapping the geographies of black-grass therefore requires a hot spot spatiotemporal imaginary that is alert to these intensities and relations: all of the folded intra-actions, multiple species, abiotic factors, and biochemical pathways that determine outbreak hot spots, as well as an attentiveness to the way these relations vary across the fields, farms, and landscapes being managed.

These regions, vectors, and hot spots imaginaries help our interviewees grapple with the ecological mechanics of black-grass proliferation, but we found that another is needed to understand when a black-grass outbreak crosses the threshold into the realm of pathogenesis. For this, our informants deploy a topological imaginary attuned to the nonlinear temporalities and stochastic dynamics of black-grass that dictate when and where black-grass might explode into unruly life. Here, they attend to the clues that can, to the diligent observer, portend a weed outbreak. This amounts to a spatiotemporal ontology akin to what Law and Mol (2001) described as a topology of fire: a means of knowing when latent forms will flicker into pathogenesis.

The discourse surrounding autumn versus spring cropping offers a good opportunity to see this fire imaginary at work. Julian Gold manages 800 ha of arable land in southeast England, and he won Soil Farmer of the Year in 2019. When asked why black-grass is currently so problematic, he explained that it was not so much a focus on spatial hot spots of poor soil health that can explain the rise of black-grass, but a history of changing cropping practices:

Everyone tells you you’re going to get black-grass on wet ground and all you do is drain your farm. … [But it’s] too many intense autumn rotations. Basically black-grass adapts. If you’ve got autumn rotations, which everyone had, it leaves it open for black-grass.

Both poor soil conditions and the black-grass-friendly shift to autumn cropping are due to human activity, but there is a more straightforward and tractable pathogenic causality at work in the latter. This is the fire imaginary we are describing here—the more immediate temporalities involved in pushing some ecological configuration into an event. Ben Taylor-Davies, a farmer and black-grass management consultant, also stresses this cause–effect aspect of a weed outbreak. To him, a black-grass outbreak is indivisible from the agro-ecological configurations being created by the management practices being employed around the farm:

What the farm does is how bad your black-grass gets.

Across the country, black-grass has changed from being a marginal and benign feature into one of the most troublesome pests. Its pathogenesis caught alight through the changes wrought by the industry’s transition to winter cropping (in which cereals are sown directly after harvest, instead of giving the land a fallow or grazing period). Many IPM guides, like the one produced by the Agriculture and Horticulture Development Board (a nondepartmental public body in the United Kingdom) on black-grass, stress the importance of reversing these trends to put out the fire: “If these factors encouraged black-grass, then the solution is obvious—do the opposite; sow crops later and reduce dependency on herbicides” (Moss 2017, 210).

Even once IPM practices appear to have a black-grass outbreak under control, farmers must be attuned to the signals warning about a possible future outbreak. At the Agrovista Lamport research station we visited, the presence of any black-grass was taken as a cue to persist with the most stringent protections: to keep up with diverse, low-intensity spring crop rotations. If a black-grass outbreak can flash into life on the farm in one or two growing seasons, farmers must be attuned to all the signals—the burning embers or smoldering tinder—that can tell of an impending fire. As an Agrovista (2022) research report explained, “Growers have to stick to the guidelines or risk going backwards with blackgrass control. … Before considering a return to winter wheat you have to reduce the weed seedbank to a low-enough level.”

When a black-grass germination can be considered aflame—when it becomes pathological—is also about contingent constructions of what constitutes an outbreak that is sufficiently problematic or risky to warrant intervention. Whether something is on fire is as much a function of personal and cultural ideas (about risk, danger, and farm business management) and farm economics as it is of ecological dynamics (about seeding rates, cultivation practices, and the soil conditions that lead to or frustrate black-grass growth). For David Purdy, an agricultural machinery merchant conducting extensive PhD research on black-grass germination explained, the development of a black-grass control program has to incorporate these other-than-ecological factors:

So there’s that tension between controlling black-grass and penalizing yourself with reduction in yield. If we can get a crop in at the end of September, that’s great, but then you stand a huge risk of running into black-grass.

Here, the flickering of a black-grass germination into the fire of a “disease situation” (Hinchliffe et al. 2016) is configured by the networks, regions, and hot spots we described earlier—but it also incorporates a more complex sociocultural calculus around finance, risk, and reward.

To summarize, our interviewees are developing a polymorphic spatiotemporal imaginary of a weed outbreak. They imagine black-grass as existing in bounded regional territories that can spread outward as well as travel along rhizomatic networked connections. They map how outbreaks occur in specific hot spots, whose geography is configured by the rhythms and intensities of agro-ecological relations that are conceived along different time scales. To understand how and when an outbreak might catch alight, they deploy a topology of fire that involves attuning to forewarnings, presences, and signals, and the convergence of social and ecological factors.

### Knowledge Intensification

The translation of this polymorphic spatial imaginary into practicable farm-level guidance is not simple. To manage black-grass via intervention in the agro-ecosystem as a whole requires expertise about how different biological, physical, and chemical agents intra-act, about which configurations lead to pathological proliferation, and which lead to their suppression. What does such knowledge look like and how is it produced? Our interviewees described and prescribed a process of knowledge intensification that they felt was required to bridge the gap between the relational ontology undergirding the configuration model of pest management (described earlier) and their biopolitical enactment (described later). In this section, we present three related components of this process of knowledge intensification: an epistemological shift from scientific reductionism to holism, a methodological shift from basic to applied research on pest management practices, and a sociological shift in the knowledge economies associated with IPM expertise.

Agrarian historians and political economists have traced how contemporary, modern, Western food production systems became defined by “input substitutions” (Altieri 1995): the replacement of labor-intensive and ecological farm management practices by applications of agricultural chemicals (pesticides and fertilizers) and the use of agricultural machinery (till, sow, spray, reap). They have shown how these substitutions were enabled by an agronomic research agenda predicated on a type of scientific reductionism (Worster 1977) that focuses on atomistic parts within systems (crops, pests, nutrients) rather than taking those systems in their integrity (Baars and Baars 2007).

This reductionist agenda has helped create a “herbicidal assemblage” geared toward chemical methods of control (Werner, Berndt, and Mansfield 2022). Akin to the Pasteurian model of biomedicine, pest management research focused on developing pesticides with “modes of action” that intervene in “target sites” to destroy specific pests (Casida 2009). This epistemological paradigm was cognate with a contamination-based approach to weed management: identifying, isolating, and targeting organisms to ensure they are absent from healthy space and its more desirable forms of life. Many reports on the barriers to greater uptake of IPM, including those related directly to black-grass management (Moss, Perryman, and Tatnell 2007), grapple with IPM’s incompatibility with this research agenda.

As Guthman (2019) traced in her exploration of California strawberry cultivation, this reductionist scientific model, along with the pest management strategies it has produced, is beginning to creak under the pressures emanating from the alterations it has made into farmland ecosystems. As Julian Gold, who we heard from earlier, put it:

[Black-grass] is like nature giving us the middle finger and saying “you think you’re so clever.” How about going back to your A-Level [high school] biology textbooks to work out that if you have systems that aren’t conducive to staying on top of weeds, they’re going to get the upper hand.

In response, black-grass experts are turning to forms of systems thinking, noting how crops, cultivation practices, and soil management solutions can be stitched together as component parts of a dynamic agro-ecological system as a means of combating black-grass (Lutman et al. 2013). As such, IPM implies a profound epistemological shift (Wolff 2023) from scientific reductionism to a more holistic mode of systems thinking.

This shift is closely related to the second feature of the knowledge intensification processes associated with configurational approaches to pestilent life: a methodological reorientation of the scientific practices used to generate disease management strategies. As Scott (1998) showed in his *Seeing Like a State,* over the course of the twentieth century, mainstream agronomic science gravitated toward basic research methods owing to the way they meshed with state and large-scale bureaucratic capitalists’ desire for *techne*—knowledge that is “impersonal, universal and completely impervious to context” (Scott 1998, 320)—and aversion to *mētis—*the “practical skills and acquired intelligence” (Scott 1998, 313)—that is embodied and context specific. Stephen Moss (or Dr. Black-grass, as he is affectionately known) traced a parallel history for us in reflecting on more than forty years of involvement in agronomic research and extension.

He explained how a growth in research on the “basic” disease mechanisms of plant pests has come at the expense of “applied” methods. He suggests that contemporary agronomic research and development has tended to focus on the specific growth mechanisms of pests like black-grass and the possible action sites for herbicidal intervention, rather than on the whole agro-ecosystem whose complex behaviors can only be effectively studied through field-level applied approaches. He noted:

We’re dealing with a very practical field problem, you know, you can’t replicate this in a lab.

This difference between basic and applied research is similar to what medical researchers describe as the difference between “bench” and “bedside” research (Woolf 2008). Moss suggests that there is too much bench research and not enough bedside research translating information into workable agricultural practice. As this gap grows, it creates a “valley of death” (Seyhan 2019): a pathological situation created by a paucity of insight into how to manage disease “out there” in the messy complexities of the world; a situation exacerbated by the tendency for technological and pharmaceutical pipelines to dry up through lack of investment and take-up.

**Figure 3.**
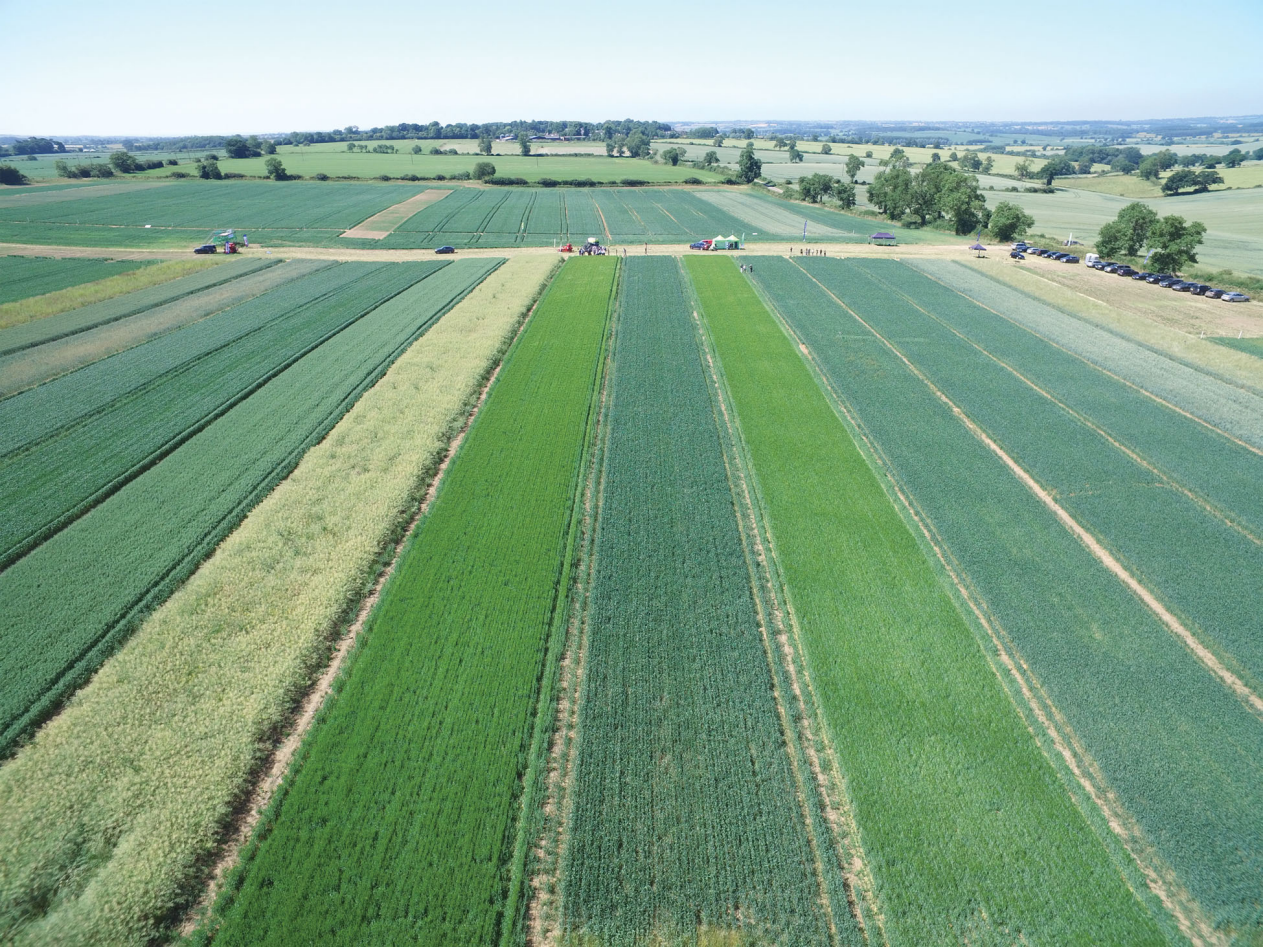
Agrovista black-grass research station. The strips each represent a longitudinal study of different approaches to black-grass management. *Source:* Images reproduced with permission from Agrovista UK, Ltd.

Those producing IPM agronomic research and offering IPM extension services aim to bridge this bench-to-bedside gap. The Agrovista research station, shown in Figure 3, is illustrative of this type of translational research. It is situated in a region with a high black-grass burden and mimics the activities of a working farm. Here, researchers conduct longitudinal and open-ended studies, charting the black-grass growth rates associated with different IPM strategies. Their methods intervene in the farm’s ecological configurations (rather than the black-grass itself) in ways that mirror the responses farmers might make to an actual outbreak. They collate and compare information on the input costs, crop yield, profits, and pest outcomes of different management inputs. Rather than develop strategies reliant on specialist machinery or proprietary chemical products, they experiment with crop rotations, tillage dates, tillage depths, cover crops, biodiversity provisioning, and so on to produce wieldy and applied insight for their farming customers.

A vital dimension of this bench-to-bedside research is its contextual specificity, capturing how different management interventions yield different results in different places with different configurations of soil type, rainfall quantity, cropping systems, historical pesticide regimes, and so on. The process of IPM knowledge intensification situates investigations at the interface of disease and place, recognizing that the conditions that lead to pathogenesis cannot be understood in absolute terms but only as a function of a plant’s relationships with its environmental conditions. Health and disease become “a matter of geographical specificity” (Hinchliffe 2022a, xxx).

This need for holistic, applied, and context-specific knowledge practices has led to a shift in the sociology of agronomic knowledge production and dissemination. This is the third feature of the knowledge intensification process associated with the configurational mode. Farm managers are learning that off-the-shelf IPM black-grass management strategies need to be tailored to the specifics of their farm’s ecological makeup. Research and agronomy firms, like Agrovista, know that this contextualization needs to be an important part of their commercial offer. As Mark Hemmant, one of their researchers, explained:

Rotational plowing definitely can reduce grass weeds, but it can make it worse. Applying that in some situations works whereas in others, it doesn’t. It’s trying to, what’s the word, interpret it for the given conditions.

There is also a highly do-it-yourself and collegiate bent to this experimentalism. The farmers we interviewed explained how they had trialed different sowing dates, tillage depths, break crops, and livestock-arable integration arrangements, and studied which practices control black-grass establishment rates in specific locations. As with the related regenerative agricultural movement (Cusworth, Garnett, and Lorimer 2021a), they share IPM knowledge and experience. They do this particularly via social media, which has become an important channel for farmer-to-farmer communication (Skaalsveen, Ingram, and Urquhart 2020). Social media is accessible on the go, it offers informal spaces where farmers feel comfortable sharing experiences, and where they can plow their social energy into groups with common political, social, and farming perspectives. Speaking in relation to crop rotations and pest control, John Cherry, a farmer and leading figure in the United Kingdom’s regenerative movement, explained:

The whole computer thing has allowed this to happen … suddenly you’ve got the ability to talk in real time to people doing extraordinary things all over the world … and you say, well, that’s exciting, we’ll try it.

Although the transfer of knowledge from one farmer to another entails the inevitable loss of context-specific resolution, the pooling of expertise and experience is nevertheless valuable for refining IPM interventions for black-grass management. The focus on context specificity and translational and applied research has also given rise to new pest management experts. These agronomists help navigate the explanatory gaps between general IPM best practice (e.g., that a transition to spring cropping can help reduce black-grass) and the application of those ideas in specific contexts. These actors are like vets in livestock systems (Hinchliffe and Ward 2014; Holloway 2019). Through situated knowledge of their client’s agro-ecological environments, they translate and interpret configurational biosecurity principles into farm-relevant insight.

### Probiotic Agribiopolitics

In this section, we trace how the polymorphic spatial imaginary and the intensive knowledge framework introduced in the previous two sections inform a probiotic approach to pest management. This approach is founded on a growing skepticism toward the antibiotic myth of hygiene and a subsequent recalibration of its modes of managing life.

This shift in thinking is not being driven by a softening of attitudes toward black-grass. Farmers certainly do not like the weed and, if they could, they would have it removed from their landscapes altogether. Their multispecies relations are still hostile and violent and organized in service of the anthropocentric goals of crop growth and farm business profit. Knowing that the goal of pest extirpation is impossible (or at least only fleetingly achieved), though, farmers and IPM experts are developing new biopolitical strategies for making live and letting die. These strategies are reconciled to the inevitability of living alongside awkward organisms like black-grass that have the potential for pathological proliferation, so long as they remain below the tolerable levels of abundance. In IPM, these are specified as *thresholds*.

Building on Lorimer (2020), we here describe an emerging “probiotic” model of biopolitics where “life is used to manage life.” These work toward the prevention and suppression of disease through interventions that modulate the configuration of agro-ecological relations on the farm. We describe two strategies in which farmers use cultural rather than chemical controls.

**Figure 4.**
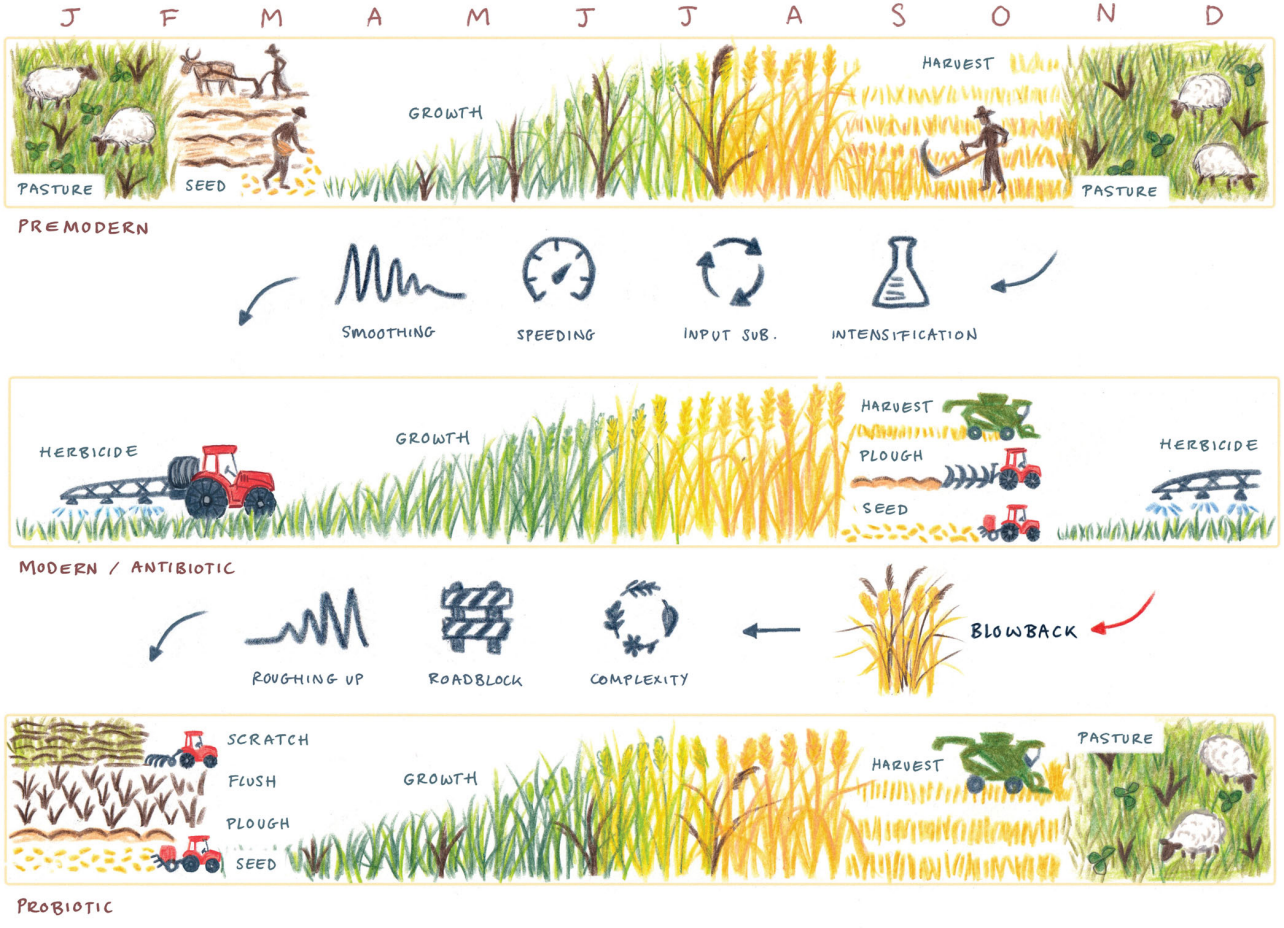
Pathogenesis metanarrative: Annual farming practices associated with a premodern regime; a modern/antibiotic model; and the probiotic regime embodied in integrated pest management black-grass management strategies. Note, this image was produced to reflect temperate agricultural settings. *Source:* Original image by Vivien Martineau.

In the first, farmers change the choreography of the agricultural calendar to modulate the sequencing of the reproductive cycles of desired and undesired plants (as shown in Figure 4). Farmers and researchers are developing cropping and cultivation systems that set aside time in the agricultural calendar for black-grass to flourish. As Tom Sewell, a farmer interviewee explained:

If we let black-grass grow, when we drill the wheat we get better control because we’ve killed out some of the flushing black-grass. Probably not all of it but a big chunk of it. And then we’ve just now [November] scratched the wheat stubbles, to flush the black-grass which has now been killed off. We still got more black-grass coming through … then just before Christmas we might go again and just give it another blast and try and get a third flush in January, February, March and then just start the oats into that and then spray again to try and get as much of the seed bank gone as we can, then make the most of spring crops.

Tom explained how he “scratches” the earth, creating a light and shallow disturbance of the topsoil to encourage black-grass to burst into life in a “flush” of vitality. These flushes can then be buried via plowing, or, in no-tillage systems, killed off with a broad-spectrum herbicide like glyphosate. Such practices create space for desired crops to be grown later in the season without its competition. Black-grass’s life force is effectively truncated into one or a small number of short and intense periods by coaxing it into life with specifically designed soil disturbance interventions. A spring crop can then (hopefully) be grown absent the pest. This involves a finely tuned choreography of the dynamics and intensities of the farm’s ecological relations over time and space. These interventions—which now circulate widely in the farming press (Farmers Weekly 2023)—orchestrate syncopated periods in which different plants, even awkward ones, are encouraged to grow.

There is a tension when attempting to manage black-grass through no-tillage regimes and the fostering of improved soil health. No-tillage relies on the herbicide glyphosate (the generic chemical name for Roundup) as farmers cannot bury their weeds with a plow. Its use is associated with a hotly contested set of downstream environmental and human health impacts, however. These trade-offs are made all the thornier by the way large agricultural chemical producers have seized on the market opportunity manifest in promoting glyphosate as an essential tool for sustainable food producers (Müller 2021). Such soil health, biodiversity, and productivity tensions amount to an archetypal case of the “multifaceted nature” (Mansfield et al. 2023) of the politics of pesticide usage. Although we do not have room to pursue these discussions further (but we direct readers to Werner, Berndt, and Mansfield [2022] and Müller [2021] for their excellent treatments), the point is that efforts to pursue probiotic biopolitical strategies to live well with awkward life like black-grass are fraught with compromise.

These trade-offs notwithstanding, our farmers make time for IPM, emulating Puig de la Bellacasa’s (2015) description of care in agricultural soil management. This injunction carries a dual meaning: Farmers must make time to attune themselves and be affected by the ecological configurations that pertain to pathogenesis. They must also make time to accommodate the multiple temporalities that define when weed management becomes an emergent, immanent feature of a farm’s agro-ecosystem. Time, in other words, is central to the means of living alongside awkward life. For these reasons, understanding whether some agroecological configuration is “immuno-competent” (Hinchliffe et al. 2013) cannot be apprehended from a single snapshot of the farm. It needs to be understood as a function of the more-than-human, abiotic, physical, chemical, and messy intra-actions that take place over longer temporal stretches.

In a second related probiotic strategy, IPM experts and farmers seek to use the ecological agencies of some organisms to prevent or suppress the growth of black-grass. They aim to “use life to manage life” (Lorimer 2020) in ways akin to how those involved in rewilding seek to restore degraded ecosystems through the introduction of keystone species like beavers, wolves, earthworms, and soil microbes. IPM black-grass management strategies are defined by an effort to foster health through the presence, not the absence of life. Farmers are finding that certain plants (like arable crops, cover crops, grasses, legumes) and animals (cows, sheep) are effective at managing pests in ways that chemical and physical controls are not. Here, ecologically significant lifeforms are deployed as agents for biocontrol. For example, livestock animals are used to improve soil conditions, improve ecological functions, and increase drainage to resist black-grass proliferation (Cusworth et al. 2022). They also enhance land-use diversity on the farm system by opening up opportunities for grass-arable rotations. As a farmer, Julian Gold, explained:

[Black-grass] is a function of our specialized systems [where animals are reared separately from arable farms]. In the old-fashioned days with rotational leys [where grass was grown in between arable crops] … you could get on top of black-grass. If you put it down to grass for like four years, then plow it up, you will probably get rid of your black-grass.

These strategies tell of an overarching probiotic strategy: to rebuild ecological abundance, diversity, and complexity on the farm with a view to enhancing the resilience of the system to withstand pest shocks. The new choreography of planting we traced earlier, for example, slows down and roughs up the seasonal cycles that were smoothed and accelerated by input substitutions, the rise of continuous cropping, and the loss of alternating arable-grass phases. Figure 4 captures how IPM and related regenerative and agro-ecological practices like crop rotation diversification and livestock arable-integration work to (re)install space–time complexity into the farm system.

In this probiotic configurational mode, there is a reluctantly pragmatic (rather than enthusiastically normative) tolerance for awkward life: an acceptance that commensals like black-grass have been made (relationally) pathological by the simplified ecological configurations created by anthropogenic activity, that their imperviousness to chemical controls is an outcome of their historical and profligate usage, and that mitigating against the power and likelihood of proliferation (rather than the total removal of life that might become pathological) is the only remaining avenue for agricultural action. All this serves to complicate the biopolitical rubric of making live and letting die. These farmers are not seeking to kill awkward life like black-grass. They are, instead, making it live in specific ways and at specified points in the agricultural calendar to ensure it does not spoil the livability of the landscapes for the crops being grown.

If pathological events and pest outbreaks are understood to be features of an agro-ecosystem as a whole rather than the proliferation of dangerous life contaminating healthy space, then their prevention demands intervention in configurations rather than the targeting of pathological bodies. There is good sense, from this vantage point, in working toward the mutual livability of awkward and sought-after bodies and bad sense in seeking to wholly exterminate the former for the benefit of the latter. Although there is still a violence involved in focusing agricultural care toward some lives and not others (Cusworth 2023), this (agri)biopolitics does not necessitate the de facto death of pestilent life.

## Conclusions: A Metanarrative of Pathogenesis in Arable Agriculture

We conclude by recapitulating the analysis we have offered here in more general and historical terms. We do this to flag its broader significance for the geographies of agricultural disease. We suggest that the case of black-grass and IPM can be used to conceptualize a chronology of the rise of probiotic approaches to tackling the “blowback” (Wallace and Wallace 2015) caused by the excessive application of antibiotic modes of managing nonhuman life. We offer the following illustration to capture this pathogenesis metanarrative.

Drawing on data-driven evolutionary models, Wallace and Wallace (2015) argued that the diversity inherent in premodern agriculture acted as a metaphorical “sterilising temperature,” curtailing the evolution and the spread of pathogenic disease, particularly novel zoonotic viral strains. They proposed that the variety of microbes, plants, and animals on such farms, cycled around over space and across time, created an agro-ecological configuration that provided evolutionary roadblocks that inhibited opportunities for pathogenic proliferation. In contrast, they suggested that the simplicity inherent in intensive farming systems drives “selection for greater virulence and lowered response to biomedical intervention” (Wallace and Wallace 2015, 2068).

Through an analysis of the pathologies of intensive agriculture, they referred to the (unforeseen) outcomes of these phase shifts as “blowback.” Black-grass provides a perfect example. Premodern farming acted as a safeguard against the rupture of black-grass outbreaks by producing configurations hostile to proliferation. Simplified, intensive and modern systems, in contrast, have reduced the friction of land-use diversity and are creating ecological blowback in the form of rapidly proliferating, herbicide-resistant black-grass populations.

The changes in farm management that drive black-grass blowback are indicative of an antibiotic mode of agriculture that seeks to simplify, smooth, and accelerate the temporal cycles of farming to make ecosystems better fit with market conditions. In the case of black-grass, the rise of continuous cropping, the loss of fallowing and grass-arable cycles, and the use of herbicides have driven increases in its herbicidal resistance and general abundance levels. These shifts degrade (at least with regard to black-grass management) the agro-ecological configuration and push the farm system over the pathogenesis threshold.

The long-standing yet marginal presence of black-grass in the places where it is now problematic is an important part of this story. It hints at the incompleteness of a model of health and disease organized around bounded regional areas contaminated by breaches of pathogenic life. Black-grass evolved alongside premodern agriculture, where it was a commensal and largely undisruptive force. It has long been here, and has certainly not invaded from out there. As such, its contemporary pathogenesis and subsequent biopolitical intervention can only be understood in terms of immanence, relations, and configurations. It differs markedly, for example, from the story of Gamba grass, which has become pathogenic in Australia as a result of intercontinental translocation into ecologies in which it lacks natural predators (Atchison and Head 2013). The point to draw from the endemic quality of the black-grass is not that configurational approaches must be reserved for dealing with pests created within a given agricultural milieu, nor that they cannot work for introduced species in novel and unprepared environments. The point, rather, is to demonstrate the explanatory deficiencies of an understanding of health and disease overly focused on breaches and contamination.

The three plot points in this metanarrative should be read as an incremental and accretive rather than epochal account of change. It is not so much that there were (good) premodern farmers managing fertility and pests through diversity who suddenly became (bad) antibiotic practitioners (Latour 1993). Even in the most intensive and chemically reliant systems, rotations remain an important part of crop protection programs, and “alternative” farming systems—like no-till, organics, and integrated pest control programs—have emerged in symbiosis with, rather than strict alterity to, commercial systems and productivity-minded governmental policy (Tomlinson 2008). Nevertheless, change has happened, and this is our attempt to essentialize a set of messy and never-comprehensive dynamics of change into a more tractable story of premodern, antibiotic, and probiotic modes.

This article has traced how an agro-ecological blowback event (like herbicidal-resistant black-grass) presents socioeconomic opportunity for creative experimentation with alternative modes of ecological governance. IPM—and other related probiotic agricultural models like regenerative farming and agro-ecology—has emerged from these experimentations. It satisfies a need for more sophisticated spatiotemporal imaginaries to conceive of agro-ecological relations, more intensive knowledge practices to understand situated and holistic ecological interactions, and it demonstrates the potential of probiotic models of biopolitics that use life to manage life. IPM intervenes into agro-ecological configurations to modulate evolutionary and biochemical processes to secure or restore tempered modes of multispecies conviviality.

These strategies have the potential to deliver relative increases in ecological functionality, biodiversity, and abundance. They stay with the trouble (Haraway 2016) of the violence inherent to agricultural production and, in the words of Tsing and her colleagues, they strive for pragmatic “arts of living on a damaged planet” (Tsing et al. 2017).
